# The Moderating Power of Impulsivity: A Systematic Literature Review Examining the Theory of Planned Behavior

**DOI:** 10.3390/pharmacy10040085

**Published:** 2022-07-18

**Authors:** Lindsey A. Hohmann, Kimberly B. Garza

**Affiliations:** 1 Department of Pharmacy Practice, Harrison College of Pharmacy, Auburn University, 1330J Walker Building, Auburn, AL 36849, USA; lah0036@auburn.edu; 2 Department of Health Outcomes Research and Policy, Harrison College of Pharmacy, Auburn University, 4306B Walker Building, Auburn, AL 36849, USA

**Keywords:** theory of planned behavior, impulsivity, behavioral economics, health behaviors, systematic review, intention–behavior gap, health behavior theories

## Abstract

The theory of planned behavior (TPB) states that behavioral intention is the best predictor of actual behavior change. However, intention explains only a portion of the variance in behavior. Of specific interest is the question of which moderating or mediating variables can be leveraged to aid health promotion interventions utilizing the tenets of behavioral economics (delay discounting and commitment contracts) in the intention–behavior pathway. Impulsivity has been postulated to fill this role and may be applied to multiple behaviors. We aim to determine if impulsivity moderates the association between intention and actual behavior in the TPB, to discover what other variables may moderate or mediate this association, and to apply the findings to future studies in the field of behavioral economics. To this end, a systematic review was conducted using the PubMed, PsychINFO, and Embase online databases. Eligible studies in peer-reviewed journals published prior to November 2021 were selected. Thirty-three studies were included in the final review, examining physical activity, diet, preventive health, mental health, addiction, and medication adherence behaviors. Three main concepts emerged: (1) impulsivity moderates the association between intention and behavior change; (2) self-efficacy moderates the association between intention and behavior change; and (3) planning and self-efficacy contribute to moderated mediation. This review demonstrates a gap in the literature regarding the application of the TPB to the intention–behavior pathway for health behaviors. Future studies in behavioral economics may leverage the variables of impulsivity, self-efficacy, and planning to predict follow-through in this area and to develop targeted change initiatives.

## 1. Introduction

The classic theory of planned behavior (TPB) states that behavioral intentions are the best predictors of actual behavior change [1,2,3]. Support for the traditional TPB is found throughout the literature, most often using cross-sectional surveys, pre-post surveys, or small timeseries [4,5,6,7,8]. The bulk of the research has been within the psychology of eating and drinking behavior, as well as physical and sexual activity, gambling, and smoking, where intention has been shown to mediate the association between behavioral perceptions and behavior change [5,6,9,10,11,12,13]. Variables of behavioral perception, including attitude towards behavior, subjective norms, and perceived behavioral control, have accounted for a range of 40 to 77% of the variance in subjects’ intentions to change behavior [10,14,15,16]. Attitude towards behavior is defined as a positive or negative individual view of that behavior, while subjective norms are defined as views or behaviors that are generally accepted or expected in the community or by personally important individuals [14]. Similarly, perceived behavioral control is defined as an individual’s belief regarding their ability to change a particular behavior [14]. Furthermore, behavioral intention has been shown to account for 15 to 39% of the variance in actual behavior change [9,15]. This fluctuation may be partially explained by variation in the topic of study (smoking, condom use, etc.) [10,15], but this does not answer the question of what accounts for the remaining variance in actual behavior change. Is intention to change behavior the only mediator between perceptions and behavior change, and is intention alone really a good predictor of behavior change? Is intention itself dependent on some other variable?

This question leads us to consider factors that may influence the relationship between intention to change behavior and actual behavior change. It seems likely that there is some additional variable that mediates (is in the causal pathway between intention and behavior change) or moderates (influences the direction and strength of the intention–behavior relationship) this process [17], especially as we take into consideration the concept of behavioral economics, which combines principles of economics and cognitive psychology and states that people do not always behave rationally [18]. Furthermore, based on the authors’ previous research [19,20], it also seems likely that this intention–behavior pathway is moderated by a level of impulsivity, where impulsivity is a broad concept containing four general domains: urgency, lack of premeditation, sensation seeking, and lack of perseverance [21]. In the realm of behavioral economics, impulsivity is defined as the tendency to place a much higher value on immediate rewards compared to rewards received in the future and is commonly operationalized using various binary, choice-discounting tasks [22]. One might imagine that, even with a strong intention to change behavior, the powerful influence of impulsive choice (or difficulty delaying gratification) can undermine attempts at actual behavior change. One might intend to go to the gym regularly, but when tempted by the prospect of sleeping in or engaging in more pleasurable sedentary behaviors, that intention may not result in frequent gym attendance. Outside of the behavioral economics literature, impulsivity has also been found to moderate the relationship between eating traits and body mass index [23], as well as between negative life events and suicidal behavior [24]. Although impulsivity, thus, seems a likely culprit, the influences of other variables cannot be discounted.

Investigating moderators and mediators of the intention–behavior pathway is, thus, critical in informing future interventions to promote beneficial health behavior and improve health outcomes. With this belief in mind, we begin to pursue leads on potential mediators and moderators of the intention–behavior gap by conducting a short literature review across a wide variety of health behaviors with the goal of informing future studies in the field of behavioral economics.

## 2. Methods

A systematic literature review was performed using the PubMed, PsychINFO, and Embase online databases according to the Preferred Reporting Items for Systematic Reviews and Meta-Analyses (PRISMA) guidelines [25]. The searches were conducted in October 2021, and entries began broadly with “theory of planned behavior”, then were progressively narrowed by adding or modifying terms, with search strings combined using the Boolean operator “OR”. The search strings included “‘Theory of planned behavior’ and intention and behavior and (moderator or mediator)”, “(Impulse or impulsiv*) and mediator and intention”, “(Impulse or impulsiv*) and behavior and moderator and intention”, “Intention and behavior and (Impulse or impulsiv*) AND ‘theory of planned behavior’.” The database hits were then combed through by title and abstract for the most relevant studies, with those mentioning novel mediators and moderators selected for full-text review. Google Scholar was also used with a “snowballing” method, in which additional papers were identified from manually searching the text and reference sections of papers selected from database hits. 

Eligibility was determined by two reviewers (LAH and KBG) in an unblinded and standardized manner. The primary author (LH) conducted title and abstract reviews, followed by full-text reviews. Full-text reviews were then independently conducted by a second reviewer (KBG), and discrepancies were resolved via discourse and consensus. Studies written in English and utilizing an experimental, quasi-experimental, or observational design in peer-reviewed journals published prior to November 2021 were included in this review. The variables sought included novel, participant-level moderators or mediators between intention and behavior change in the theory of planned behavior. In order to provide a foundation for future interventions across a variety of disciplines within behavioral economics, studies examining a broad range of health behaviors were eligible for this review. Studies were excluded if they were: unclear whether moderation or mediation was being tested (not explicitly stated by the authors or unable to distinguish by methods); not examining the theory of planned behavior; or not testing the moderation or mediation of variables between intention and a health behavior change. Moderating or mediating variables controlling for study procedures were not of interest. EndNote version X9 (ClarivateTM, Philadelphia, PA, USA) citation management software was used as a data organization tool and to assist with removal of duplicate articles. 

The data were extracted by one investigator (LH) using a standardized template, including study design, intervention setting and population, study period, health behavior, measure scales used, statistical significance (yes or no), and risk of bias. Risk of bias (ROB) was assessed within each study using the mixed methods appraisal tool (MMAT) version 2018 [26]. This tool is suitable for a wide variety of study designs and consists of a 5-item measure of methodological quality (e.g., *“Is the sample representative of the target population?”*) rated as “Yes” (quality criteria are met), “No” (quality criteria are not met), or “Can’t Tell” (not enough information to determine whether quality criteria are met). The quality scores were calculated based on the number of items rated “Yes”, with a score of 0–1 being low quality (high risk of bias), 2–3 being moderate quality (moderate risk of bias), and 4–5 being high quality (low risk of bias) [27]. The findings were independently confirmed by a second reviewer (KBG), with discrepancies resolved via discourse and consensus. Due to the heterogeneous nature of behavioral intervention studies, the results were not pooled, nor were meta-analytic methods used. No registration was filed for this protocol. 

## 3. Results

### 3.1. Database Hits

**Figure 1:**
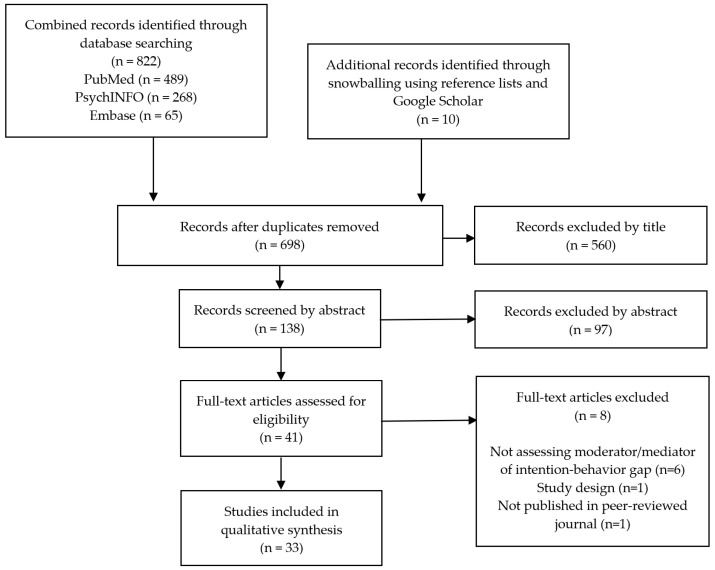
PRISMA Diagram. Database hits and stepwise inclusion or exclusion of studies.

**Figure 2:**
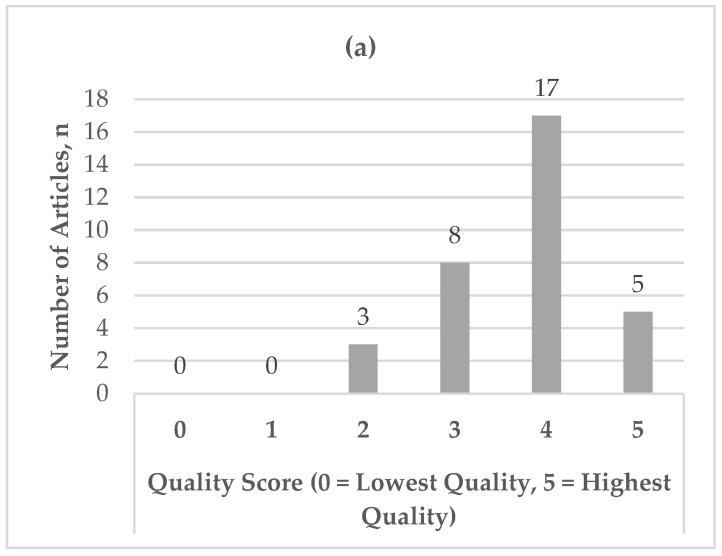

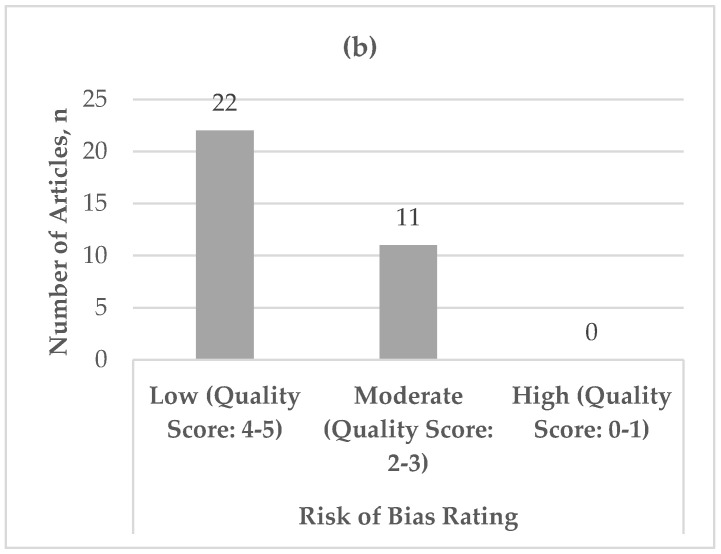
(**a**,**b**) Risk of bias assessment using mixed methods appraisal tool (MMAT): (**a**) frequency of article quality scores in risk of bias assessment (*n* = 33) and (**b**) frequency of article risk of bias ratings (*n* = 33).

The initial search yielded 822 hits, with 698 after the removal of duplicates (Figure 1). After title and abstract review, 41 full-text articles were assessed for eligibility, and 33 studies met the inclusion criteria and were retained in the final review [16,21,28,29,30,31,32,33,34,35,36,37,38,39,40,41,42,43,44,45,46,47,48,49,50,51,52,53,54,55,56,57,58]. The majority of the studies utilized a noncontrolled timeseries or pre-post design, with follow-up periods ranging from 1 week to 6 months (Table 1). No significant conflicts of interest were noted amongst the studies, with the risks of bias rated as low (*n* = 22) or moderate (*n* = 11) (Figure 2a,b). The behaviors of interest included physical activity (*n* = 17), diet (*n* = 9), preventive health behaviors (e.g., condom use or sun protection; *n* = 7), addiction (*n* = 6), mental health (*n* = 3), and medication adherence (*n* = 1) (Table 2a). Most articles reported moderation analyses (*n* = 30), followed by mediation (*n* = 5), moderated mediation (*n* = 4), and moderated moderation (*n* = 3), with more than one type of analysis or variable of interest per study in some cases (Table 2b). The specific moderating and mediating variables surrounding the intention–behavior gap are presented below.

### 3.2. Moderators and Mediators with a High Level of Support 

**Figure 3:**
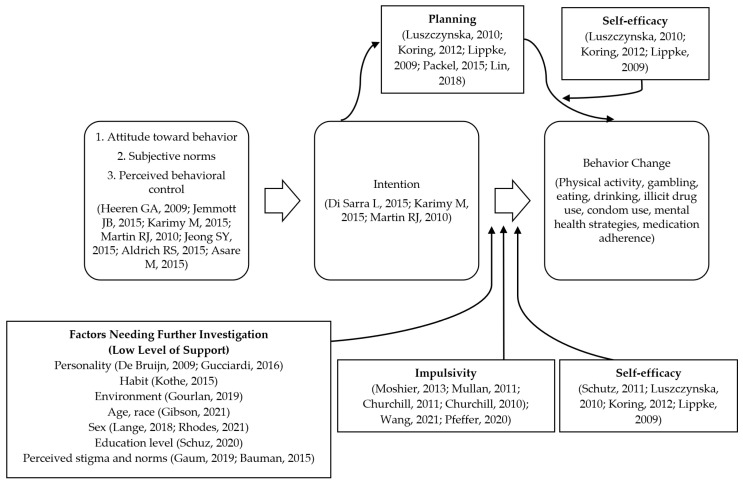
Summary of statistically significant literature review findings as applied to an extended view of the theory of planned behavior [5,6,9,10,11,12,14,15,16,21,29,30,31,32,36,37,38,39,40,43,44,45,52,53,54,57].

Sixteen articles assessing moderators and mediators of the intention–behavior gap contributed to a straightforward picture of variables’ effects, with the majority of these studies (*n* = 12) reporting statistically significant findings and limited contradictory findings (Table 2c and Figure 3).

#### 3.2.1. Impulsivity Moderates the Association between Intention and Behavior Change

Six studies showed that impulsivity moderates the relationship between behavioral intention and actual behavior change (Table 1 and Table 2c, and Figure 3) [21,28,29,30,31,32]. These results spanned several disciplines, most notably substance misuse and addiction, diet, and physical activity. The common theme of these studies was that the intention to change behavior leads to an actual behavior change, but the magnitude of this change depends on the level of impulsivity in the individual. Specifically, Moshier and colleagues studied the effect of impulsivity on illicit drug use and found that premeditation and sensation seeking were moderator variables affecting the pathway between the intention to stop drug use and actual drug use in the previous month [21]. Similarly, Mullan et al. found that higher inhibitory control and planning ability, two elements implicit in the concept of impulsivity, resulted in less binge drinking in college students with the intention to drink [30], and Wang [32] found that lower problem-gambling severity (a proxy for impulsivity) led to less unintended sports-gambling behavior. This moderation was echoed by the findings of Churchill [28,29], who demonstrated greater decreases in unintended snacking [28] and increases in intended fruit and vegetable consumption [29] when urgency (or impulsivity) was lower. Furthermore, Pfeffer and colleagues [31] found that self-control moderated the path between intentions and physical activity, such that higher self-control resulted in increased physical activity in those intending to exercise. 

Although the majority of the articles demonstrated support for impulsivity as a moderator, three studies assessed the moderating role of impulsivity in the intention–behavior gap but found no statistical significance [33,34,35]. Specifically, Chevance and colleagues’ [33] investigation of physical activity, Crandall’s [34] assessment of mindfulness mobile app usage, and Stevens’ [35] evaluation of alcohol consumption did not demonstrate a significant association. 

#### 3.2.2. Self-Efficacy Moderates the Association between Intention and Behavior Change

Moderation was not only shown to occur via impulsivity, but self-efficacy demonstrated a moderation effect as well (*n* = 4; Table 1 and Table 2c, and Figure 3) [16,36,37,38]. Schutz and colleagues showed that HIV-positive men who have sex with men were approximately 21 times more likely to act on their intentions to use condoms when they had higher self-efficacy [36]. Self-efficacy was defined in this case as the perceived ability to surmount barriers that impede the fulfillment of a particular behavior [36,59]. In this way, self-efficacy is related to the concept of perceived behavioral control, or one’s belief in the ability to affect change in a particular behavior [14,60]. Similarly, Koring [37], Lippke [38], and Luszczynska [16] all found that intentions to be physically active were put into practice more often in those with higher versus lower self-efficacy. On the other hand, C.Q. Zhang and colleagues [41] assessed the moderating roles of action self-efficacy (perceived ability to start a behavior) and maintenance self-efficacy (perceived ability to continue a behavior in the face of barriers) [61] on hand-washing and sleep hygiene behaviors, but no statistically significant moderating effects were found. Further pursuit of this thread led to a more complex view of self-efficacy as a part of moderated mediation in the context of an additional variable: planning. 

#### 3.2.3. Planning Mediates the Association between Intention and Behavior Change

Five studies demonstrated a statistically significant mediating effect of planning in the intention–behavior pathway (Table 1 and Table 2c, and Figure 3) [16,37,38,43,44]. Four of these studies focused on behaviors related to physical activity [16,37,38,44]. Koring [37], Luszczynska [16], Lippke [38], and Packel [44] found that the intention to exercise translated into actual physical activity indirectly via planning. While Koring [37] assessed planning in general, other studies have broken planning down into its constituent parts of action planning (making plans to act on intentions) and coping planning (making plans to overcome expected barriers) [61]. Specifically, Luszczynska [16] and Packel [44] found that both action planning and coping planning filled this mediating role, while Lippke [38] found statistical significance for action planning alone. In terms of behaviors other than physical activity, Lin and colleagues [43] found that the intention to adhere to a medication influenced actual medication adherence (to aspirin) indirectly via both action planning and coping planning. However, when planning was viewed in combination with self-efficacy, a more complicated picture of moderated mediation emerged. 

#### 3.2.4. Planning and Self-Efficacy Contribute to Moderated Mediation

Moderated mediation between intentions and behavior change was demonstrated via planning and self-efficacy in several studies (*n* = 3; Table 1 and Table 2c, and Figure 3) [16,37,38]. Moderated mediation occurs when a variable mediates the relationship between the behavioral intention and the behavior change, and an additional variable acts as the moderator of this process. Koring [37], Luszczynska [16], and Lippke [38] and colleagues independently found that planning mediated the pathway from intentions to actual changes in physical activity. Individuals’ levels of self-efficacy moderated this relationship; in other words, higher self-efficacy increased the likelihood of plans being realized as actual changes in physical activity behavior [16,37,38]. This was a more complicated process than noted by Schutz’s [36], Lin [43], and Packel [44] and opened the consideration of additional moderators and mediators within the theory of planned behavior and the intention–behavior gap, specifically. 

### 3.3. Moderators and Mediators with Less Support 

Seventeen articles reported moderators and mediators of the intention–behavior gap that were less clear, with fewer statistically significant findings (*n* = 10) or mixed findings, which warrant additional investigation in future studies (Table 2c and Figure 3). 

#### 3.3.1. Personality Moderates the Association between Intention and Behavior Change

Of seven studies assessing personality traits as moderators in the intention–behavior pathway, two studies found statistically significant moderators [45,46], while five studies did not [47,48,49,50,51] (Table 1 and Table 2c, and Figure 3). De Bruijn et al. [46] assessed neuroticism and conscientiousness, which are two traits from the Five-Factor Model of Personality (FFM), and found that neuroticism moderated the intention–fruit consumption gap, such that lower neuroticism led to a stronger relationship between intention and behavior. Additionally, Gucciardi [45] found mental toughness to be a statistically significant moderator, resulting in increased translation of exercise intentions into actual exercise performance. On the other hand, MacCann [47] and Monds [48] assessed the moderating effect of the HEXACO personality domains (honesty and humility, emotionality, extraversion, agreeableness, conscientiousness, and openness to experience) on the intention–physical activity and intention–fruit and vegetable consumption pathways, respectively, and found no statistically significant moderators. Similarly, Hannan [50] and Hartson [51] found no moderating roles for mental toughness or mindfulness, respectively, between intention and physical activity, while Cao [49] found that mental toughness served as a moderator in the intention–physical activity gap in Chinese college students but not in adult wage earners. 

#### 3.3.2. Socioeconomic Factors Moderate the Association between Intention and Behavior Change

Four studies assessed moderation by socioeconomic factors (*n* = 4 statistically significant), with a wide variety of factors assessed, including age, race, sex, and education level (Table 1 and Table 2c, and Figure 3) [52,53,54,55]. Gibson [53] found that age and race moderated between the intention to social distance and actual social distancing behavior during the COVID-19 pandemic, such that intentions were translated into practice more often in older adults and those identifying as White. Additionally, Schuz [54] found that a higher education level resulted in a greater influence of intention on multiple different behaviors, including fruit and vegetable consumption, physical activity, alcohol consumption, flossing, healthy snack consumption, limiting the amount of time spent sitting daily, low-fat diet, and breast or testicular self-exams. Lange and colleagues [55] examined the role of sex in a moderated mediation model and found that sex moderated the effect of planning in the intention–behavior gap in cases of fruit and vegetable consumption, physical activity, and sun protection behaviors, with planning mediating the intention–behavior pathway for men but not women. On the other hand, Rhodes [52] found a moderating effect of sex within the intention–physical activity pathway, such that intention was successfully translated into physical activity for women but not men. However, when the perceived opportunity for exercise was added to the model, physical activity intentions were successfully realized for men more than women (moderated moderation). With the limited number of studies focused on each socioeconomic factor, it was difficult to draw conclusions about any particular factor. 

#### 3.3.3. Perceptions and Beliefs Regarding Stigma and Norms Moderate the Association between Intention and Behavior Change

Two studies found particular perceptions or beliefs that moderated the intention–behavior gap (Table 1 and Table 2c, and Figure 3) [56,57]. Specifically, Gaum and colleagues [56] found that lower anticipated stigma resulted in the increased implementation of depression prevention strategies in the workplace. Likewise, Bauman [57] demonstrated that those with higher normative belief incongruence (changes in normative beliefs over time) were more likely to consume alcohol despite the opposite intention. However, multiple studies assessing perceived stigma or belief incongruence are necessary in order to come to a more firm conclusion regarding their roles as moderators. 

#### 3.3.4. Environment Moderates the Association between Intention and Behavior Change

The evidence was mixed concerning the moderating effect of environment on the intention–behavior gap, with one study finding a statistically significant effect [58] and one study finding no effect [39] (Table 1 and Table 2c, and Figure 3). Gourlan and colleagues [58] found that built environment, when combined with planning, contributed to moderated moderation of the intention–physical activity gap, such that those with a greater level of planning and living closer to exercise facilities were more likely to follow through with intentions to exercise. However, R. Zhang [39] found that perceived worksite neighborhood walkability had no moderating role between the intention to walk and actual physical activity. 

#### 3.3.5. Habit Moderates the Association between Intention and Behavior Change 

Evidence was also mixed for the moderating role of habit (Table 1 and Table 2c, and Figure 3) [40,42]. Specifically, Kothe [40] found that self-reported habit strength increased follow-through on the intention to adhere to a gluten-free diet. On the other hand, Allom et al. [42] found that habit strength did not serve to moderate the intention–physical activity gap. 

## 4. Discussion

The bulk of our findings regarding additional variables in an extended version of the theory of planned behavior suggested the moderation of the intention–behavior pathway by impulsivity [21,28,29,30,31] or self-efficacy [16,36,37,38], mediation by planning [16,37,38,43,44], and some evidence for moderated mediation via planning and self-efficacy [16,37,38]. However, the evidence for moderation by other personality and demographic traits, such as neuroticism, age, and gender, was sparse [46,48,53], with a lack of consistent measurement instruments or definitions for personality traits. Furthermore, there was a paucity of research investigating habit, built environment, and stigmatizing and normative beliefs within the intention–behavior gap, as well as research evaluating the interactions of these variables with impulsivity. These findings align with previous systematic reviews focused solely on physical activity and sun protection behaviors, which have found some evidence for the moderation of the intention–behavior gap by self-efficacy but have shown mixed evidence for sociodemographic variables [62,63]. Future studies assessing the moderators and mediators between intention and behavior should focus on the aforementioned variables, which have a current low level of support in the literature. 

When taken as a whole, these studies showed the broad application of the theory of planned behavior throughout various disciplines. Studies investigating physical activity and dietary behaviors were most prominent, as well as substance misuse, mental health, condom use, and other preventive health behaviors. However, using our protocol and inclusion criteria, only one study was found that addressed patient medication adherence. One can imagine that medication-taking behavior is highly related to the level of impulsivity, given that many medications for chronic diseases have immediate costs, including adverse effects, while the benefits of taking them are often not apparent until sometime in the future. Indeed, studies have shown that certain character traits, including impulsivity, predict low medication adherence [64]. However, patient medication adherence remains low despite educational interventions targeting patients and providers [65]. Thus, this is a critical area for future study. 

### 4.1. Implications

This review has implications for future studies in the field of behavioral economics and health outcomes. The concepts of impulsivity and planning are especially relevant to this field, as the theoretical framework of the discipline relies on these individual characteristics. Commitment contracts (financial or social incentives to perform a behavior to which individuals agree ahead of time) in behavioral economics are particularly relevant to the role of planning as a mediational variable. Studies have shown that the use of financial commitment contracts, where participants’ previously deposited money is deducted for not meeting a behavioral goal, are able to overcome high levels of impulsivity to increase follow-through in the desired behavior [66]. Reward substitution, or the provision of short-term financial or social incentives to avoid engaging in an undesirable behavior, has also been used successfully to overcome difficulty in delaying gratification [67]. Future studies may include pre-post or small timeseries surveys examining the role of impulsivity as a moderator between patient intentions and health behaviors. Follow-up studies may use commitment contracts, incentives, or decision aids as a means to investigate behavior follow-through in highly impulsive participants. Further investigation also needs to be conducted on the effects of age and gender in the intention–behavior pathway; if effects are found, this may be significant for the development of targeted and tailored health behavior tools or interventions. The pursuit of such studies can help to fill the knowledge gap demonstrated by this review and may have implications for patient and prescriber incentives in the increasingly outcome-oriented United States healthcare system. 

### 4.2. Limitations

Although this systematic review was performed following the PRISMA guidelines [25], the results must be interpreted with caution in light of several limitations. First, the majority of the studies exploring this topic utilized cross-sectional, pre-post, or small timeseries designs with no more than 6 months of follow up, which may not be sufficient time to effect or maintain a behavioral change, limiting the validity of the findings. However, given the psychological nature of many of these studies, these designs were the most ethical and feasible. 

Second, the heterogeneity of the study designs and behaviors studied limited the ability to pool the findings. This precludes the use of meta-analytic methods. However, the findings may still be clinically valuable and informative for behavioral interventions. 

Third, studies using the theory of reasoned action (TRA) were not included in this review. The TRA is closely related to the TBP, but the TPB includes one additional construct-perceived behavioral control. In order to focus the review effort, only studies examining the TPB were included; however, the inclusion of “TRA” in the search parameters may have yielded additional studies in other areas of health behavior. This may be included in future studies. However, a preliminary search did not yield appreciably different results. 

Fourth, varying definitions and measurements of impulsivity were found amongst the studies. Where the definitions were unclear, the reviewers’ judgement was used; however, this may limit the replicability of the search. Furthermore, planning was found to be a mediational variable in several studies, yet planning may also be related to the concept of impulsivity or self-regulation [30], making this association unclear. Additionally, in some cases, it was difficult to determine whether moderation or mediation was being tested if it was not explicitly stated by the authors.

## 5. Conclusions

The bulk of the evidence from this review pointed to impulsivity as a moderator between intention and behavior change, with a possible moderating role of self-efficacy. Furthermore, planning, which has been defined in several studies as a composite of both action planning and coping planning [68,69], may play a mediational role between behavioral intention and actual behavior change. However, despite the breadth of the application in topics of psychology, this review demonstrated a gap in the literature regarding the study of moderators and mediators in the theory of planned behavior as it applies to behavioral economics and health outcomes. Future studies assessing moderators and mediators between intentions and health behavior should focus on personality, habit, built environment, stigmatizing and normative beliefs, and sociodemographic variables, which have a current low level of support in the literature. 

## Figures and Tables

**Table 1:** Final study characteristics.

Article Author, Year	Study Design	Intervention Population	Follow-Up Period	Behavior of Interest	Moderating or Mediating Variables (Scales)	Statistical Significance (*p*-Value)
Allom, 2016 [42]	Noncontrolled timeseries (pre-post)—T1, T2	*n* = 101 Australian college students Mean age: 19.60 yrs Female: 81.40%	1 week	Physical activity	Moderation	
					Habit strength (12-item Self-Report Habit Index)	No
Baumann, 2015 [57]	Noncontrolled timeseries (pre-post)—T1, T2	*n* = 433 German adult job agency clients Mean age: 30.6 yrs Female: 36%	3 months	At-risk alcohol use	Moderation	
					Belief incongruence	
					○ Normative belief incongruence (4-item measure)	Yes (<0.05)
					○ Behavioral belief incongruence (6-item measure)	No
					○ Control belief incongruence (4-item measure)	No
Cao, 2021 [49]	Noncontrolled timeseries (pre-post)—T1, T2	*n* = 591 Chinese college students Mean age: not reported (range 19–24 yrs) Female: 57.53%; and *n* = 285 Chinese adult wage earners Mean age: not reported (range 27–58 yrs) Female: 44.56%	1 week	Physical activity	Moderation	
					Mental toughness (8-item Mental Toughness Inventory (MTI))	
					○ Among college students	Yes (<0.01)
					○ Among wage earners	No
Chevance, 2018 [33]	Noncontrolled timeseries (pre-post)—T1, T2	*n* = 76 French adults Mean age: 56 yrs Female: not reported	4 months	Physical activity	Moderation	
					Trait impulsivity (20-item UPPS-P Impulsive Behavior Scale)	No
					○ Lack of conscientiousness	No
					Executive functions (Computerized Wisconsin Card-Sorting Test)	No
Churchill, 2010 [28]	Noncontrolled timeseries (pre-post)—T1, T2	*n* = 256 UK adults Mean age: 33.05 yrs Female: 79.36%	2 weeks	Avoidance of snacking	Moderation	
					Impulsivity at T2 (45-item UPPS Impulsive Behavior Scale)	
					○ Urgency	Yes (<0.05)
					○ Lack of premeditation	No
					○ Lack of perseverance	No
					○ Sensation seeking	No
Churchill, 2011 [29]	Controlled timeseries—T1, T2, T3	*n* = 323 UK adults Mean age: 32.8 yrs Female: 81.42% Students: 52.94%	2 weeks (1-week intervals)	Fruit and vegetable consumption	Moderation	
					Impulsivity at T1 (45-item UPPS Impulsive Behavior Scale)	
					○ Urgency	Yes (<0.05)
					○ Lack of premeditation	No
					○ Lack of perseverance	No
					○ Sensation seeking	No
Crandall, 2019 [34]	Noncontrolled timeseries (pre-post)—T1, T2	*n* = 85 Undergraduate college students in Utah, US Mean age: 21.81 yrs Female: 53%	2 weeks	Mindfulness meditation mobile app use	Moderation	
					Executive functioning (three tasks from the NIH Toolbox (NIH-TB)	No
					○ Cognitive shifting (NIH-TB Dimensional Change Card-Sort Test)	No
					○ Inhibitory control and attention (NIH-TB Flanker Inhibitory Control and Attention Test)	No
					○ Working memory (NIH-TB List-Sorting Working Memory Test)	No
De Bruijn, 2009 [46]	Cross-sectional interviewer-administered survey	*n* = 405 Dutch adults Mean age: 60.25 yrs Female: 57.53%	No follow-up	Fruit consumption	Moderation	
					Five-Factor Model of Personality (FFM)	
					○ Neuroticism (6-item measure)	Yes (<0.001)
					○ Conscientiousness (6-item measure)	No
Gaum, 2019 [56]	Cross-sectional survey	*n* = 112 German adults with history of depression Mean age: 42.3 yrs Female: 75%	No follow-up	Implementation of depression prevention strategies at work	Moderation	
					Anticipated stigmatization (2-item German Inventory of Subjective Stigma Experience)	Yes (0.003)
					Experienced stigmatization (2-item German Inventory of Subjective Stigma Experience)	No
Gibson, 2021 [53]	Noncontrolled timeseries (pre-post)—T1, T2	*n* = 507 US adults Mean age: 50.39 yrs Female: 50.9%	3 months	Social distancing during COVID-19	Moderation	
					Age	Yes (<0.001)
					Race	Yes (0.002)
					Intention stability over time	Yes (<0.001)
Gourlan, 2019 [58]	Noncontrolled timeseries (pre-post)—T1, T2	*n* = 219 French adults Mean age: 41.28 Female: 52.51%	3 months	Physical activity	Moderation	
					Planning (5-item French Action-Planning Scale)	No
					Perceived built environment (3-item measure)	No
					Moderated Moderation	
					Planning*Environment	Yes (0.02)
Gucciardi, 2016 [45]	Noncontrolled timeseries (pre-post)—T1, T2	*n* = 193 Australian adults Mean age: 30.79 yrs Female: 55.44%	2 weeks	Rehabilitation exercises for knee pain	Moderation	
					Mental toughness (8-item index)	Yes (0.013)
Hannan, 2015 [50]	Noncontrolled timeseries (pre-post)—T1, T2	*n* = 117 Australian adults and undergraduate students Mean age: 28.29 yrs Female: 73.50%	1 week	Physical activity	Moderation	
					Mental toughness (8-item Mental Toughness Index (MTI))	No
Hartson, 2020 [51]	Noncontrolled timeseries (pre-post)—T1, T2	*n* = 232 US Hispanic adolescents Mean age: 15.23 yrs Female: 51.3%	2 weeks	Physical activity	Moderation	
					Mindfulness (10-item Child and Adolescent Mindfulness Measure)	No
Koring, 2012 [37]	Noncontrolled timeseries—T1, T2, T3	*n* = 290 German adults Mean age: 41.9 yrs Female: 77%	6 weeks	Physical activity	Moderation	
					Self-efficacy (2-item measure)	Yes (<0.05)
					Mediation	
					Planning (2-item measure)	Yes (<0.05)
					Moderated Mediation	
					Planning*Self-efficacy	Yes (<0.05)
Kothe, 2015 [40]	Cross-sectional survey	*n* = 228 Australian adults with Celiac disease Mean age: 45.2 yrs Female: 89.5%	No follow-up	Gluten-free diet adherence	Moderation	
					Perceived behavioral control (PBC) (17-item TPB Celiac Disease Questionnaire)	No
					Habit (12-item Self-Reported Habit Index)	Yes (0.013)
					Moderated Moderation	
					PBC*Habit	Yes (<0.001)
Lange, 2018 [55]	Study I. Noncontrolled timeseries—T1, T2, T3	*n* = 461 German adults Mean age: 38.2 yrs Female: 81.6%	4 months	Fruit and vegetable intake	Moderated Mediation	
					Planning*Sex (3-item planning measure)	Yes (0.040)
	Study II. Noncontrolled timeseries—T1, T2, T3	*n* = 193 German university students Mean age: 24.5 yrs Female: 80.8%	2 weeks	Physical activity	Planning*Sex (4-item planning measure)	Yes (0.022)
	Study III. Noncontrolled timeseries (pre-post)—T1, T2	*n* = 166 German adults Mean age: 37.6 yrs Female: 49.3%	2 weeks	Sun protection	Planning*Sex (2-item planning measure)	Yes (0.014)
Lin, 2018 [43]	Noncontrolled timeseries (pre-post)—T1, T2	*n* = 535 Iranian women with high-risk pregnancies Mean age: 32.29 yrs Female: 100%	8 weeks	Medication adherence (aspirin)	Mediation	
					Planning	
					○ Action planning (4-item measure)	Yes (<0.01)
					○ Coping planning (5-item measure)	Yes (<0.01)
Lippke, 2009 [38]	Noncontrolled timeseries (pre-post)—T1, T2	*n* = 812 German adults Mean age: 36.69 yrs Female: 74.4%	4 weeks	Physical activity	Moderation	
					Self-efficacy (3-item measure)	Yes (<0.01)
					Mediation	
					Action planning (3-item measure)	Yes (<0.01)
					Moderated Mediation	
					Action planning*Self-efficacy	Yes (<0.01)
Luszczynska, 2010 [16]	Study I. Noncontrolled timeseries (pre-post)—T1, T2	*n* = 534 Chinese adolescents, grades 7–12 Mean age: 13.8 yrs Female: 54%	4 weeks	Physical activity	Moderation	
					Self-efficacy (3-item scale)	Yes (<0.01)
					Mediation	
					Coping planning (4-item measure)	Yes (<0.01)
					Moderated Mediation	
					Coping planning*Self-efficacy	Yes (<0.01)
	Study II. Noncontrolled timeseries (pre-post)—T1, T2	*n* = 620 Polish high school adolescents Mean age: 16.46 yrs Female: 62%	10 weeks	Physical activity	Moderation	
					Maintenance of self-efficacy (7-item measure developed via elicitation study)	Yes (<0.05)
					Mediation	
					Action planning (5-item measure)	Yes (<0.05)
					Moderated Mediation	
					Action planning*Self-efficacy	Yes (<0.05)
MacCann, 2015 [47]	Cross-sectional survey	*n* = 1017 US college students Mean age: 23.1 yrs Female: 63.9%	No follow-up	Physical activity	Moderation	
					Personality (6 HEXACO personality domains measured via 96-item International Personality Item Pool (IPIP))	
					○ Honesty and humility (16 items)	No
					○ Emotionality (16 items)	No
					○ Extraversion (16 items)	No
					○ Agreeableness (16 items)	No
					○ Conscientiousness (16 items)	No
					○ Openness to Experience (16 items)	No
Monds, 2016 [48]	Cross-sectional survey	*n* = 1036 US college students Mean age: 23.08 yrs Female: 63.9%	No follow-up	Fruit and vegetable consumption	Moderation	
					Personality (6 HEXACO personality domains measured via 96-item International Personality Item Pool (IPIP))	
					○ Honesty and humility (16 items)	No
					○ Emotionality (16 items)	No
					○ Extraversion (16 items)	No
					○ Agreeableness (16 items)	No
					○ Conscientiousness (16 items)	No
					○ Openness to Experience (16 items)	No
Moshier, 2013 [21]	Cross-sectional survey	*n* = 84 Adults receiving methadone maintenance treatment from 2 outpatient clinics in Boston, US Mean age: 40 yrs Female: 56%	No follow-up	Illicit drug use	Moderation	
					Impulsivity (44-item UPPS Impulsivity Scale)	
					○ Urgency	No
					○ Lack of premeditation	Yes (0.015)
					○ Lack of perseverance	No
					○ Sensation seeking	Yes (0.007)
Mullan, 2011 [30]	Noncontrolled timeseries (pre-post)—T1, T2	*n* = 153 Australian university students Mean age: 20.1 yrs Female: 73.86%	1 week	Binge drinking of alcohol	Moderation	
					Impulsivity and Self-Regulation (Executive Function measures)	
					○ Planning ability (the Tower of Hanoi task)	Yes (0.03)
					○ Inhibitory control (the Stroop Task)	Yes (0.035)
					○ Decision making (the Iowa Gambling Task)	No
					○ Cognitive flexibility (the Wisconsin Card-Sorting Task)	No
Packel, 2015 [44]	Cross-sectional survey	*n* = 96 Adults with colorectal cancer in Pennsylvania, US Mean age: 65.6 yrs Female: % not reported	No follow-up	Physical activity	Mediation	
					Planning	
					○ Action planning (4-item Action-Planning and Coping-Planning Scale—Physical Exercise)	Yes (0.007)
					○ Coping planning (5-item Action-Planning and Coping-Planning Scale—Physical Exercise)	Yes (0.001)
Pfeffer, 2020 [31]	Noncontrolled timeseries (pre-post)—T1, T2	*n* = 118 University students Mean age: 24.74 yrs Female: 70.3%	4 weeks	Physical activity	Moderation	
					Trait self-control (13-item Brief Self-Control Scale)	Yes (0.033)
Rhodes, 2021 [52]	2-arm parallel randomized trial (groups collapsed)—T1, T2, T3, T4	*n* = 254 Canadian adults who were new parents Mean age: 31.94 yrs Female: 50%	Baseline, 6 weeks, 12 weeks, 6 months	Physical activity	Moderation	
					Gender	Yes (<0.01)
					Moderated Moderation	
					Gender*Action control (M-PAC model)	
					○ Gender*Affective attitude	No
					○ Gender*Perceived opportunity	Yes (<0.05)
					○ Gender*Planning	No
					○ Gender*Habit	No
					○ Gender*Identity	No
Schutz, 2011 [36]	Noncontrolled timeseries (pre-post)—T1, T2	*n* = 237 HIV-positive men who have sex with men in Montreal, Canada Mean age: 42.5 yrs	6 months	Condom use	Moderation	
					Self-efficacy (11-item interview measure)	Yes ^a^
					Perceived behavioral control (3-item interview measure)	No
					Past behavior (3-item interview measure)	No
					Moral norm (3-item interview measure)	No
					Anticipated regret (3-item interview measure)	No
					Role beliefs (3-item interview measure)	No
					Sociodemographics	No
					Context	No
					Life experience	No
Schüz, 2020 [54]	Study I. Cross-sectional survey	*n* = 1005 US adults Mean age: 33.6 yrs Female: 47.1%	No follow-up	1. Fruit and vegetable consumption 2. Physical activity 3. Low-fat diet 4. Alcohol consumption 5. Flossing daily 6. Testicular or breast self-exams	Moderation	
					Socioeconomic status (SES)	
					○ Education level (categorical multiple choice based on the US Census Current Population Survey and International Standard Classification of Education (ISCED))	Yes (<0.05)
					○ Income (categorical multiple choice)	No
					○ Occupation status (percentage unemployment level matched to zip code based on American Community Survey; area-level SES measure)	No
					○ Zip code (text entry; area-level SES measure)	No
					○ Subjective SES (10-point ladder subjective SES scale)	No
	Study II. Noncontrolled timeseries (pre-post)—T1, T2	*n* = 1273 International adults Mean age: 31.57 yrs Female: 50.5%	4 weeks	1. Fruit and vegetable consumption 2. Physical activity 3. Alcohol consumption 4. Flossing daily 5. Not sitting for extended periods 6. Healthy snack consumption	Moderation	
					Socioeconomic status (SES)	
					○ Education level (categorical multiple choice based on the US Census Current Population Survey and International Standard Classification of Education (ISCED))	Yes (<0.01)
					○ Income (categorical multiple choice)	No
					○ Occupation status (personal employment)	No
					○ Subjective SES (10-point ladder subjective SES scale)	Yes (<0.05)
Stevens, 2017 [35]	Noncontrolled timeseries (pre-post)—T1, T2	*n* = 77 US young adults Mean age: 20.8 yrs Female: 60.5%	10 days	Alcohol consumption	Moderation	
					Impulsivity	
					○ Lack of planning (59-item UPPS-P Impulsive Behavior Scale)	No
					○ Lack of perseverance (59-item UPPS-P)	No
					○ Negative urgency (59-item UPPS-P)	No
					○ Positive urgency (59-item UPPS-P)	No
					○ Sensation seeking (59-item UPPS-P)	No
					○ Response inhibition (Go–Stop Impulsivity Paradigm)	No
					○ Response initiation (Immediate memory Task (IMT))	No
					○ Delay discounting (27-item Monetary Choice Questionnaire (MCQ) and Two-Choice Impulsivity Paradigm (TCIP))	No
Wang, 2021 [32]	Cross-sectional survey	*n* = 334 US college students. Mean age: 21 yrs Female: 32.3%	No follow-up	Sports gambling	Moderation	
					Problem-gambling severity (4-item scale)	Yes (0.003)
Zhang C.Q., 2020 [41]	Noncontrolled timeseries—T1, T2, T3	*n* = 297 College students in China Mean age: not reported (range: 18-35 yrs) Female: 82.49%	2 months	Hand washing and sleep hygiene	Moderation (intention—hand washing)	
					Action planning (3-item scale)	Yes (<0.001)
					Coping planning (3-item scale)	No
					Action self-efficacy (3-item scale)	No
					Maintenance self-efficacy (3-item scale)	No
					Moderation (intention—sleep hygiene)	
					Action planning (3-item scale)	No
					Coping planning (3-item scale)	No
					Action self-efficacy (3-item scale)	No
					Maintenance self-efficacy (3-item scale)	No
Zhang R., 2019 [39]	Noncontrolled timeseries (pre-post)—T1, T2	*n* = 157 Office employees in China Mean age: 33.26 yrs Female: 64.97%	1 month	Transport-related walking	Moderation	
					Worksite neighborhood walkability index (abbreviated Neighborhood Environment Walkability Scale (Chinese NEWS-A))	No

^a^ *p*-value not reported. * Variables interact within moderated mediation or moderated moderation analyses.

**Table 2:** (**a**) Number of articles describing a particular behavior, (**b**) number of articles describing moderation or mediation analyses within the intention–behavior gap, and (**c**) number of articles describing a particular moderator or mediator within the intention–behavior gap.

	(a)			
Behavior Category	Specific Behaviors Included in Category		*n*	
**Physical Activity**	General physical activity, transport-related walking, and knee pain rehabilitation exercises		17	
**Diet**	Fruit and vegetable consumption, snacking, low-fat diet, and gluten-free diet		9	
**Preventive Health Behaviors**	Flossing, hand washing, social distancing, limited sitting, condom use, breast or testicular self-exam, and sun protection		7	
**Addiction**	Alcohol use, illicit drug use, and sports gambling		6	
**Mental Health**	Mindfulness meditation app, depression prevention strategies, and sleep hygiene		3	
**Medication Adherence**	Aspirin adherence		1	
**(b)**				
**Type of Analysis**	***n***			
	**Statistically Significant**	**Not Statistically Significant**	**Total**	
**Moderation**	19	11	30	
**Mediation**	5	0	5	
**Moderated Mediation**	4	0	4	
**Moderated Moderation**	3	0	3	
**(c)**				
**Moderator or Mediator Category**	**Moderator and Mediator Variables**	***n***		
		**Statistically** **Significant**	**Not Statistically Significant**	**Total**
**More Support**	Impulsivity Moderation	6	3	9
	Self-Efficacy Moderation	4	1	5
	Planning Mediation	5	0	5
	Planning*Self-Efficacy-Moderated Mediation	3	0	3
**Less Support**	Personality Moderation	2	5	7
	Socioeconomics Moderation	4	0	4
	Perceptions and Beliefs Moderation	2	0	2
	Environment Moderation	1	1	2
	Habit Moderation	1	1	2

## Data Availability

Not applicable.
